# Human Cytomegalovirus Induces TGF-β1 Activation in Renal Tubular Epithelial Cells after Epithelial-to-Mesenchymal Transition

**DOI:** 10.1371/journal.ppat.1001170

**Published:** 2010-11-04

**Authors:** Masako Shimamura, Joanne E. Murphy-Ullrich, William J. Britt

**Affiliations:** 1 Department of Pediatrics, University of Alabama at Birmingham, Birmingham, Alabama, United States of America; 2 Department of Pathology, University of Alabama at Birmingham, Birmingham, Alabama, United States of America; 3 Department of Microbiology, University of Alabama at Birmingham, Birmingham, Alabama, United States of America; Oregon Health and Science University, United States of America

## Abstract

Human cytomegalovirus (HCMV) infection is associated epidemiologically with poor outcome of renal allografts due to mechanisms which remain largely undefined. Transforming growth factor-β1 (TGF-β1), a potent fibrogenic cytokine, is more abundant in rejecting renal allografts that are infected with either HCMV or rat CMV as compared to uninfected, rejecting grafts. TGF-β1 induces renal fibrosis via epithelial-to-mesenchymal transition (EMT) of renal epithelial cells, a process by which epithelial cells acquire mesenchymal characteristics and a migratory phenotype, and secrete molecules associated with extracellular matrix deposition and remodeling. We report that human renal tubular epithelial cells infected *in vitro* with HCMV and exposed to TGF-β1 underwent morphologic and transcriptional changes of EMT, similar to uninfected cells. HCMV infected cells after EMT also activated extracellular latent TGF-β1 via induction of MMP-2. Renal epithelial cells transiently transfected with only the HCMV IE1 or IE2 open reading frames and stimulated to undergo EMT also induced TGF-β1 activation associated with MMP-2 production, suggesting a role for these viral gene products in MMP-2 production. Consistent with the function of these immediate early gene products, the antiviral agents ganciclovir and foscarnet did not inhibit TGF-β1 production after EMT by HCMV infected cells. These results indicate that HCMV infected renal tubular epithelial cells can undergo EMT after exposure to TGF-β1, similar to uninfected renal epithelial cells, but that HCMV infection by inducing active TGF-β1 may potentiate renal fibrosis. Our findings provide *in vitro* evidence for a pathogenic mechanism that could explain the clinical association between HCMV infection, TGF-β1, and adverse renal allograft outcome.

## Author Summary

Human cytomegalovirus (HCMV) is a common virus that establishes lifelong persistence in the host. Although asymptomatic in healthy people, HCMV can reactivate and cause disease in immunosuppressed patients, such as those undergoing kidney transplantation. HCMV infection is associated with inferior renal allograft survival compared to transplants without HCMV infection. HCMV infected allografts also contain higher levels of the fibrogenic cytokine, transforming growth factor-β1 (TGF-β1), compared to uninfected allografts. TGF-β1 is a potent inducer of renal fibrosis and causes epithelial-to-mesenchymal transition (EMT), whereby epithelial cells acquire characteristics of cells of mesenchymal origin and express molecules associated with fibrosis. Our work shows that renal epithelial cells infected *in vitro* with HCMV can undergo EMT, but that HCMV infected cells produce greater amounts of the fibrogenic molecule TGF-β1, compared to uninfected cells after EMT. We have shown that this effect is likely due to specific HCMV genes (IE1, IE2), and cannot be prevented by administration of antivirals such as ganciclovir or foscarnet. These data suggest that HCMV may contribute to adverse renal allograft outcome by exacerbating TGF-β1 induced renal fibrosis. Understanding such mechanisms will permit the development of treatments that could improve long-term renal allograft survival in HCMV infected patients.

## Introduction

Human cytomegalovirus (HCMV) has been associated with poor renal allograft outcome in numerous seroepidemiologic studies [1], [2], [3], [4]. Evidence of active CMV infection (DNAemia, antigenemia) or CMV disease in renal transplant recipients is also associated with poor graft outcome [5]. In a rat renal allograft model, infection with rat CMV accelerates and intensifies rejection in infected allografts as compared to uninfected allografts [6], [7], [8]. These studies support an association between HCMV and adverse renal allograft outcome, but the mechanisms by which HCMV contribute*s* to renal allograft loss remain cryptic.

The fibrogenic cytokine transforming growth factor-β1 (TGF-β1) is present in biopsy specimens of human renal allografts undergoing rejection [9], [10], [11]. TGF-β1 is produced by infiltrating leukocytes during rejection and may also be produced by renal tubular epithelium [12], [13], [14]. TGF-β1 is expressed at higher levels in HCMV infected renal allografts compared to uninfected allografts [15]. In a rat renal transplantation model, allografts from rat CMV infected animals also contain greater quantities of TGF-β1 as compared to uninfected allografts [6], [16], [17]. TGF-β1 contributes to renal fibrosis in numerous animal models and in human fibrotic renal disease, by inducing epithelial-to-mesenchymal transition (EMT) of renal tubular epithelial cells [18], [19], [20], [21]. During EMT, renal tubular cells demonstrate loss of epithelial characteristics and cellular adhesions, develop changes in the actin cytoskeleton, induce expression of fibrogenic molecules, and acquire a migratory phenotype [22]. These fibroblastoid renal tubular cells are key contributors to renal fibrosis, as inhibition of TGF-β1 mediated EMT prevents and reverses experimentally induced renal fibrosis in animal models [22], [23], [24]. The association between CMV and TGF-β1 in renal allografts raises the possibility that CMV might accelerate renal allograft loss via viral induction of TGF-β1 with resultant fibrosis within the allograft.

Studies performed *in vitro* have shown that CMV induces secretion of TGF-β1 from infected fibroblasts, astrocytes, and osteosarcoma cells [25], [26], [27]. TGF-β1 production can also be induced by transient transfection of expression plasmids containing the HCMV immediate early 1 and 2 (IE1, IE2) genes into fibroblasts and astrocytoma cells [25], [28]. In those studies, increases in TGF-β1 were associated with induction of TGF-β1 mRNA. However, the local effects of TGF-β1 are often controlled *in vivo* by activation of the extracellular latent form [29]. Known activators of latent TGF-β1 include proteases (plasmin), matrix metalloproteases (MMPs), thrombospondin-1 (TSP-1), and the α_v_β_6_ and α_v_β_8_ integrins [30]. In the HCMV infected placenta, HCMV infected endothelial cells have been shown to induce production of TGF-β1 and collagen IV via induction of α_v_β_6_ integrin [31]. Thus, precedent exists for the possibility that HCMV infected renal cells might induce TGF-β1 production or activation in pathological settings.

HCMV can infect renal tubular epithelial cells. HCMV antigens and DNA are found in renal epithelial cells in kidneys of trauma victims examined during autopsy as well as in biopsies of renal allografts, indicating that these cells can harbor HCMV in both healthy persons and allograft recipients [32], [33]. HCMV antigens have also been detected in tubular cells of biopsies from HCMV seropositive patients with rejection [34]. Furthermore, HCMV has been detected more often in renal tubular epithelium of allograft biopsies with rejection compared to those without rejection using both immunohistochemistry and *in situ* hybridization [35], [36].

Based on the epidemiologic data associating HCMV infection with long-term allograft loss, histologic evidence that TGF-β1 production is increased in HCMV infected renal allografts, and *in vitro* data supporting HCMV induction of TGF-β1 production by fibroblasts and other cells, we hypothesized that HCMV infected renal tubular epithelium undergoing EMT might develop a fibroblast-like phenotype with secretion of TGF-β1. In the following studies, we demonstrate that HCMV infected renal tubular epithelial cells undergo EMT and thereafter induce active TGF-β1 production. In this system, MMP-2 mediates the extracellular activation of TGF-β1 in HCMV infected cells, and can be recapitulated by transient transfection of the HCMV IE1 or IE2 open reading frames into renal epithelial cells stimulated to undergo EMT. These data provide supportive evidence that HCMV infected renal epithelium may contribute to long-term renal allograft loss by exacerbating fibrosis via TGF-β1, and provides a potential *in vitro* mechanism for the association of HCMV infection with greater TGF-β1 production in renal allografts and long-term allograft loss.

## Results

### HCMV infects renal tubular epithelium

Primary human foreskin fibroblasts and the immortalized human proximal renal tubular epithelial cell line, HK-2, were infected in parallel with HCMV strain TR at an MOI of 1. Some HK-2 cultures were treated with recombinant active (ra) TGF-β1 at 15 ng/ml, starting 1 hour post-infection. Culture media and cells were harvested separately on days 1-6 post-infection and viral titers determined by the detection of early antigen fluorescent foci (DEAFF) assay. Separate cell cultures were infected and harvested at day 5 post-infection, and cellular lysates subjected to western blotting using a high titered human CMV immune globulin (Cytogam).

**Figure 1:**
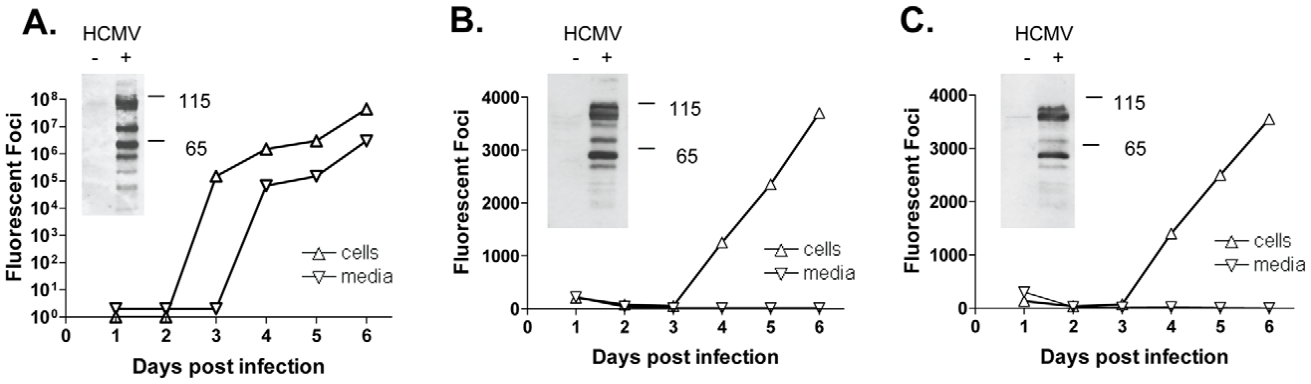
HCMV replication in HFFs and HK-2 cells. HFF (A) and HK-2 cells (B) were infected with HCMV strain TR at MOI of 1, cells and media harvested daily, and viral titers determined by DEAFF assay. A parallel set of HK-2 cells were infected with HCMV and exposed to recombinant active TGF-β1 (C), and cells and media analyzed as for HK-2 cells. A separate set of cells were uninfected or infected with HCMV strain TR at MOI of 1, cell pellets harvested at day 5 post-infection, and western blotting for viral proteins performed using HCMV hyperimmune globulin (insets). HFFs supported logarithmic viral replication with viral progeny in both cells and media, whereas HK-2 supported linear productive infection in cells only. Upright triangles, cells; inverted triangles, media.

Fibroblasts supported productive infection with logarithmic increases in viral progeny observed in both media and cells (Figure 1A). HK-2 cells also supported productive infection (Figure 1B) but the kinetic was linear and the virions remained cell-associated, with very few virions detectable in media. By day 3 post-infection, virus was not detectable in the media. No difference in viral growth kinetics was observed in the HK-2 cultures treated with TGF-β1 (Figure 1C), compared to HK-2 cultures without TGF-β1. Viral titers in HK-2 cells and media were similar at each time point post infection, in the presence and absence of TGF-β1. This result differs from TGF-β1 effects upon HFFs, where HCMV replication is induced by exposure to TGF-β1 [37]. Western blotting of the infected cell lysates (Figure 1, insets) indicates a similar pattern of infected cell viral proteins in both HFFs and HK-2 cells, consistent with productive infection in both cell types. Viral replication studies were repeated in primary renal epithelial cells and results similar to those in HK-2 cells were observed, with the majority of the replicating virus remaining cell-associated (Figure S1).

### HCMV infected primary renal tubular epithelial cells and HK-2 cells undergo TGF-β1 induced EMT *in vitro*

**Figure 2:**
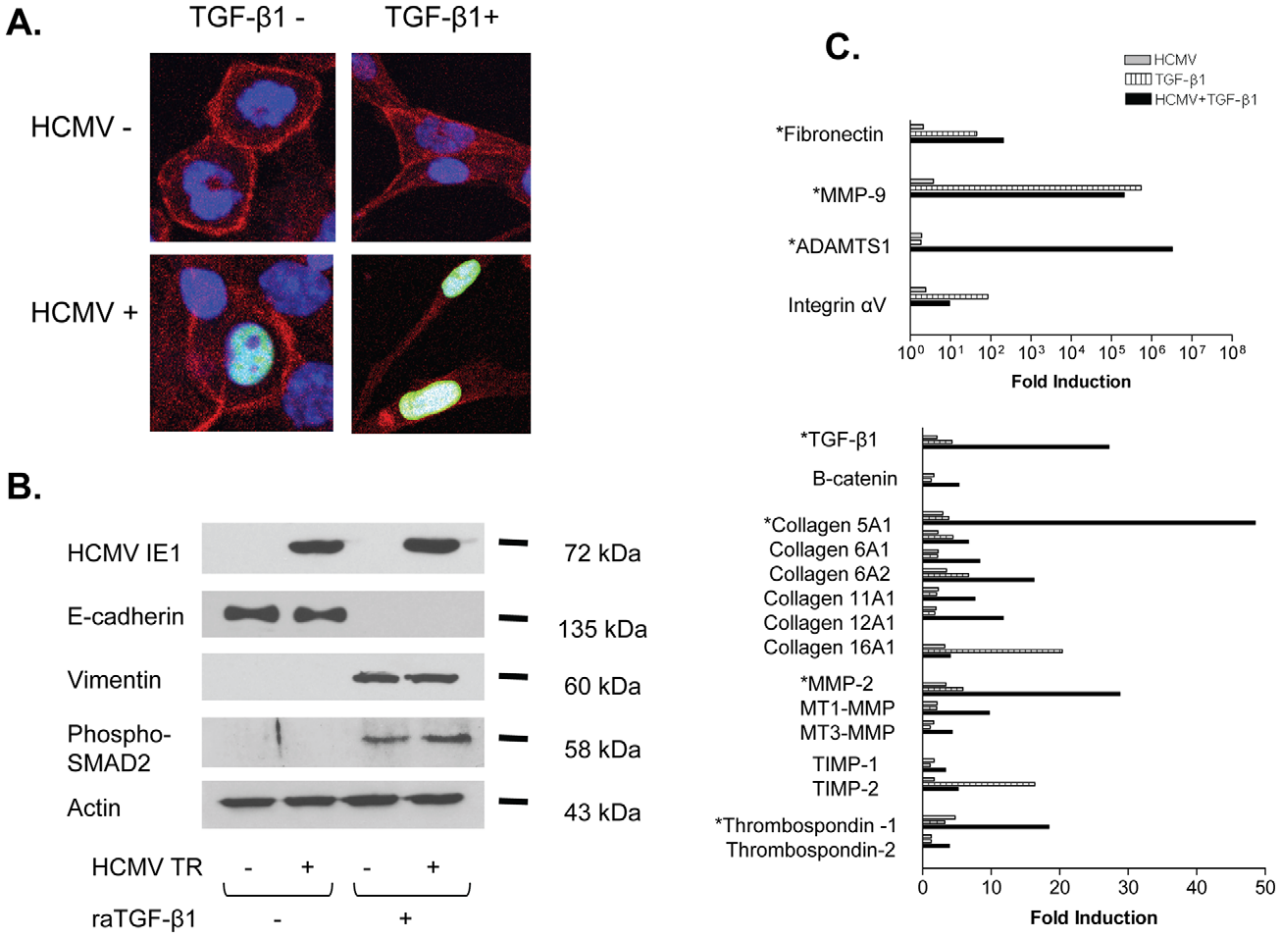
HCMV infected renal tubular epithelial cells undergo epithelial-to-mesenchymal transition (EMT) after TGF-β1 exposure. (A) Primary human renal tubular epithelial cells were uninfected (top row) or infected with HCMV strain TR (bottom row), without (left column) or with recombinant human active TGF-β1 (raTGF-β1) to induce EMT (right column). Cells were stained using a monoclonal antibody against HCMV IE1 (mab63-27) and an isotype-specific AlexaFluor 488-conjugated secondary antibody (green nuclei), and co-stained with AlexaFluor 594-conjugated phalloidin (red) and Topro3 (blue nuclei) nuclear stain. Images were collected by confocal microscopy using similar exposure time and identical gain. Cells at baseline had epithelioid morphology with concentric structural actin cytoskeleton, both without (top left) and with (bottom left) HCMV infection, whereas cells after raTGF-β1 stimulation showed elongated mesenchymal morphology indicative of EMT in both HCMV uninfected (top right) and infected (bottom right) cells. (B) Primary human renal tubular epithelial cells were untreated, or were infected with HCMV strain TR at MOI of 1 and/or treated with raTGF-β1, lysed, and subjected to western blotting using antibodies against HCMV IE1, e-cadherin, vimentin, or actin. Nuclear extracts from cellular lysates were also subjected to western blotting for phospho-SMAD2. Both uninfected (IE1 negative) and HCMV infected (IE1 positive) cells expressed E-cadherin but not vimentin at baseline, but after raTGF-β1 stimulation, both uninfected and infected cells lost E-cadherin expression and demonstrated both vimentin expression and SMAD2 phosphorylation, indicative of EMT in both. (C) HK-2 cells were untreated, or were infected with HCMV strain TR at MOI of 1 and/or stimulated with raTGF-β1 to induce EMT, lysed, total RNA reverse transcribed to cDNA, and cDNA analyzed for presence of extracellular matrix associated mRNAs using the SuperArray extracellular matrix PCR array. Results were normalized to GAPDH expression and quantitated as fold-change compared to mRNA levels in uninfected, unstimulated HK-2 cells. HCMV infected cells (grey bars) induced some mRNA transcripts of fibrogenic molecules, but only at less than 10-fold induction. Both uninfected cells stimulated with raTGF-β1 (hatched bars) and HCMV infected cells stimulated with raTGF-β1 (black bars) demonstrated induction of many fibrogenic molecules represented in this array, consistent with induction of EMT in both uninfected and HCMV infected cells. Transcripts upregulated in HCMV infected HK-2 cells after raTGF-β1 stimulation (denoted with asterisks) were confirmed by individual RT-PCR assays using both HK-2 cells and primary renal tubular epithelial cells (Figure S2).

We next investigated whether HCMV infected renal tubular cells could undergo EMT similar to uninfected cells, or whether infection caused cells to remain epithelioid in the presence of TGF-β1. HK-2 cells were infected with HCMV strain TR, followed by incubation with raTGF-β1 for 48 hours as an inducer of EMT. Cells were fixed, permeabilized, and stained using an anti-HCMV IE1 antibody (p63-27) and species-specific AlexaFluor 488 conjugated secondary antibody, and the AlexaFluor 594 conjugated phalloidin (Figure 2A). Imaging by confocal microscopy demonstrated that HCMV infected renal tubular epithelial cells exhibited cuboidal structural actin cytoskeleton, similar to uninfected cells (Figure 2A, left column). The HCMV infected renal epithelial cells appeared morphologically indistinguishable from uninfected cells and could only be distinguished by immunostaining for HCMV IE1. After raTGF-β1 stimulation, HCMV infected epithelial cells showed changes in actin cytoskeleton, developing an elongated mesenchymal phenotype associated with parallel actin stress fiber formation and loss of cuboidal epithelial morphology. The raTGF-β1 induced cytoskeletal changes appeared similar in both HCMV infected and uninfected renal tubular cells (Figure 2A, right column). Imaging results were similar using primary renal tubular epithelial cells (data not shown [dns]). These results showed that HCMV infected renal tubular cells can undergo morphologic changes consistent with EMT, similar to those described for uninfected cells [22].

In the next series of experiments, primary renal tubular cells were left untreated, or were infected with HCMV strain TR, followed by incubation with or without raTGF-β1 for 48 hours. This permitted analysis of effects of HCMV alone, TGF-β1 alone, or the combination of HCMV and TGF-β1 upon renal tubular cells. Cells were lysed and equivalent protein lysates were assayed for expression of HCMV IE1, E-cadherin, vimentin, or actin by immuno-blotting (Figure 2B). At baseline, both HCMV uninfected and infected cells expressed E-cadherin but not vimentin, consistent with an epithelial phenotype. Following raTGF-β1 stimulation, E-cadherin was no longer present, and vimentin was induced by both HCMV uninfected and infected cells, consistent with a mesenchymal phenotype. Immunoblotting for phosphorylated SMAD2 (p-SMAD2) in nuclear extracts revealed the presence of p-SMAD2 in nuclei of raTGF-β1 treated cells, both in the presence and absence of HCMV infection, whereas cells that were not treated with raTGF-β1 did not contain nuclear p-SMAD2. Again, this result was consistent with the induction of TGF-β1 dependent SMAD signaling in HCMV infected cells. HCMV infected cells expressed IE1 as expected, and actin staining showed equivalent protein loading for all samples. Taken together, these results were consistent with findings from imaging studies (Figure 2A) and indicated that HCMV infected renal tubular epithelial cells could undergo loss of E-cadherin expression, acquisition of vimentin expression, and SMAD2 phosphorylation in the presence of raTGF-β1. The phenotypic changes were consistent with changes indicative of EMT in uninfected renal tubular epithelium [22], [38].

To characterize the phenotype of HCMV infected HK-2 cells before and after EMT, HK-2 cells were examined at baseline, or after HCMV infection, in the presence or absence of raTGF-β1. Thus, effects of HCMV alone, TGF-β1 alone, or HCMV and TGF-β1 together could be compared to HK-2 cells at baseline. RNA was extracted from cell lysates, and RT-PCR performed using a commercial PCR array to detect transcripts associated with extracellular matrix molecules. Transcripts from cells under various experimental conditions were compared to those produced at baseline by uninfected, unstimulated HK-2 cells. Compared to uninfected cells, cells infected with HCMV showed less than 10-fold induction of many mRNA transcripts encoding fibrogenic matrix proteins represented in this array (Figure 2C, grey bars). This is consistent with the light microscopic appearance of HK-2 cells infected with HCMV, which appeared epithelioid and morphologically indistinguishable from adjacent uninfected HK-2 cells (Figure 2A), and confirmed that, in the absence of raTGF-β1 stimulation, HCMV infected HK-2 cells did not induce global transcriptional changes suggestive of EMT.

In contrast, after exposure to raTGF-β1, transcriptional up-regulation of a number of fibrogenic molecules was observed, indicating induction of EMT in both HCMV uninfected and infected cells (Figure 2C). Some transcripts, such as for fibronectin, MMP-9, and the α_v_ integrin, were highly upregulated in both uninfected (hatched bars) and HCMV infected (black bars) cells stimulated with raTGF-β1. A few transcripts, such as for TIMP-2, were induced to higher levels in HCMV uninfected cells compared to HCMV infected cells after raTGF-β1 stimulation. Transcripts encoding many fibrogenic molecules (ADAMTS1, TGF-β1, β-catenin, collagens, MMPs, TIMP-1, thrombospondins) were induced to greater levels in HCMV infected cells as compared to uninfected cells after EMT. These results suggested that HCMV infected renal tubular cells expressed mRNA transcripts of numerous fibrogenic proteins at similar or higher levels than uninfected renal tubular cells after induction of EMT with raTGF-β1. These results indicated that HCMV infected cells were capable of exhibiting the fibrogenic phenotype of EMT, and that HCMV infection does not reduce or prevent the EMT phenotype in these cells. To confirm the results from those studies using PCR array, individual RT-PCR assays were repeated in separate experiments using both HK-2 cells and primary renal tubular epithelial cells, utilizing commercial primer-probe sets for several of the mRNAs (Figure 2C, asterisks) that were upregulated in HCMV infected HK-2 cells after EMT (fibronectin, MMP-9, ADAMTS1, TGF-β1, collagen 5A1, MMP-2, and thrombospondin-1). These individual PCR assays validated the upregulation of these mRNA transcripts observed in the PCR array (Figure S2). The mRNAs for MMP-9 and ADAMTS1 showed a lesser log induction in the individual assays compared to the PCR array (10^3^–10^4^ fold induction vs. 10^6^–0^7^ fold induction); however, the individual assays did confirm that these mRNA transcripts were highly upregulated after TGF-β1 stimulation, consistent with the array results, in both HK-2 cells and primary PTECs (Figure S2).

Taken together, these results demonstrate by morphologic and phenotypic assays that HCMV infected cells can undergo EMT similar to uninfected cells. HCMV infection does not prevent or diminish the EMT phenotype as compared to uninfected cells.

### HCMV infected renal tubular epithelial cells induce active TGF-β1 production after EMT

**Figure 3:**
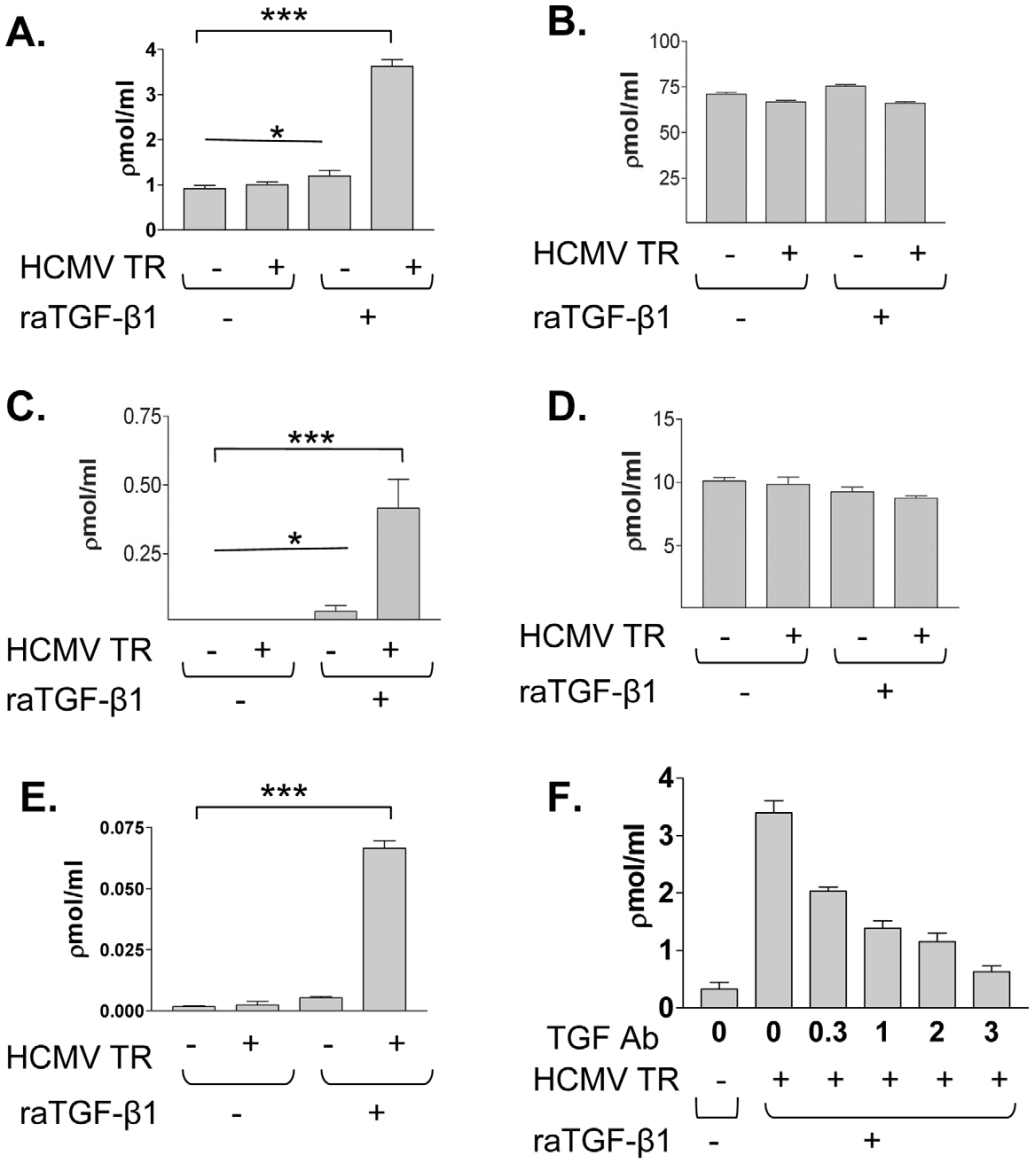
HCMV infected renal tubular epithelial cells induce active TGF-β1 production after EMT. Immortalized renal tubular cells, HK-2 (A, B), or primary human renal tubular epithelial cells (C, D) were untreated, or were infected with HCMV strain TR (HCMV TR −/+) and/or treated with raTGF-β1 (raTGF-β1 −/+) at 15 ng/ml (0.6 nM) to induce EMT. Cells were washed 3 times and re-incubated in fresh media not containing raTGF-β1. Supernatants were assayed for *de novo* active and total TGF-β1 production using a TGF-β1 responsive luciferase bioassay. Only the HCMV infected cells stimulated with raTGF-β1 induced production of active TGF-β1 in both HK-2 and primary cells. (E) HK-2 cells were infected with HCMV strain TR at MOI of 1 and/or stimulated with raTGF-β1 at 15 ng/ml as described, and supernatants were assayed using the Quantikine ELISA. Only the HCMV infected, raTGF-β1 stimulated cells produced detectable active TGF-β1 in this assay. (F) HK-2 cells were infected with HCMV strain TR at MOI of 1 and/or stimulated with raTGF-β1 at 15 ng/ml as described, and a blocking antibody against TGF-β1 was added to cell cultures simultaneously with raTGF-β1. After 48 hours, cells were washed and re-incubated in fresh media, and luciferase assay for active TGF-β1 performed. The blocking antibody reduced TGF-β1 activation in a dose-dependent manner. Legend: (*) p>0.05, ns; (***) p<0.01.

Because of the association of TGF-β1 with HCMV infection in renal allografts, we next explored whether HCMV infected HK-2 cells could produce TGF-β1. HK-2 cells were untreated, or infected with HCMV strain TR at an MOI of 1 and/or stimulated with raTGF-β1 for 48 hours to induce EMT. Cells were washed extensively to remove exogenous TGF-β1, fresh media applied and samples harvested at 24 hours after washing. Samples were assayed for active or total (active + latent) TGF-β1 activity using a published luciferase reporter bioassay (Figure 3A, B) and a commercial human TGF-β1 ELISA (Figure 3E). Because all exogenous raTGF-β1 was washed from the cultures and only fresh media not containing exogenous TGF-β1 was assayed, the TGF-β1 observed in samples was presumed to be derived from the cultured cells. No TGF-β1 was detectable in the serum-free media used for HK-2 cell culture by luciferase bioassay or ELISA (dns).

At baseline, non-infected HK-2 cells produced some detectable active TGF-β1 (HCMV TR-/raTGF-β1-) (Figure 3A). HCMV infected HK-2 cells (HCMV TR+/raTGF-β1-) also produced similar amounts of active TGF-β1 compared to uninfected cells, indicating that HCMV infection alone did not induce *de novo* TGF-β1 production in these epithelial cells (Figure 3A). This result differed from results shown by others for HCMV infected fibroblasts and astrocytes, where HCMV infection induced TGF-β1 production, and may reflect biological differences between infection of those cell types as compared to renal epithelial cells and possibly the strain of virus utilized in these previous studies [25], [26]. Uninfected HK-2 cells stimulated with raTGF-β1 (HCMV TR-/raTGF-β1+) also produced similar levels of active TGF-β1 compared to both infected and uninfected cells not exposed to raTGF-β1, indicating that EMT alone did not induce HK-2 cells to produce additional amounts of active TGF-β1 (Figure 3A). This result also confirmed that the exogenous raTGF-β1, used to induce EMT, did not carry over in detectable quantities to our assay for active TGF-β1. In contrast, HCMV infected HK-2 cells stimulated with raTGF-β1 (HCMV TR+/raTGF-β1+) produced significantly more active TGF-β1 (p≤0.01) than did cells in other conditions (Figure 3A). The quantity of total TGF-β1 was similar in all conditions (Figure 3B), indicating that changes in active TGF-β1 did not derive from an increase in latent TGF-β1. A commercial ELISA for TGF-β1 indicated that the TGF-β detected in the luciferase bioassay was TGF-β1 and not other TGF-β isoforms, and confirmed the induction of TGF-β1 in HCMV infected, raTGF-β1 infected cells (Figure 3E). The quantity of active TGF-β1 protein measured in the ELISA was approximately 3 log (1000-fold) lower than the quantity of TGF-β1 activity detected by luciferase assay. These studies were performed with equivalent volume samples obtained from the same experimental cultures, confirming that the picomolar measurements indeed differed between the two assays; however, significant induction of active TGF-β1 was measured in the HCMV infected, raTGF-β1 treated cells by both methods. These studies have not been performed in parallel by other investigators, and the differing results obtained from assays of the same samples suggests that the luciferase bioassay may detect the downstream function of a given quantity of protein more sensitively than the measurement of protein by antibody binding in the ELISA.

Together, these results suggested that HCMV infection of HK-2 cells, without EMT, did not induce *de novo* production of active TGF-β1, whereas the HCMV infected epithelial cells after EMT appeared to acquire the capacity to produce *de novo* active TGF-β1, similar to the phenotype demonstrated by HCMV infected fibroblasts [25]. Uninfected HK-2 cells, before or after EMT, did not acquire the capacity to produce *de novo* active TGF-β1.

These studies were repeated using primary renal proximal tubular epithelial cells (Figure 3C, D). These cells produced undetectable active TGF-β1 at baseline (HCMV TR-/raTGF-β1-), with HCMV infection alone (HCMV TR+/raTGF-β1-), and produced only a small amount of detectable active TGF-β1 after raTGF-β1 stimulation (HCMV TR-/raTGF-β1+), consistent with data derived from studies of HK-2 cells (Figure 3A). Similar to the HK-2 cells, HCMV infected, raTGF-β1 stimulated primary renal epithelial cells (HCMV TR+/raTGF-β1+) produced active TGF-β1 (p<0.01). The total TGF-β1 was similar in all treatment conditions, consistent with results from HK-2 cells (Figure 3D). These results indicated that the induction of active TGF-β1 production observed in HCMV infected HK-2 cells after EMT was not unique to immortalized cells such as HK-2 but was likely a general phenotype observed for both immortalized and primary renal tubular epithelial cells after HCMV infection.

Next, HK-2 cells were infected with HCMV at an MOI of 1, stimulated with raTGF-β1 at 15 ng/ml, and incubated with a function blocking antibody against TGF-β1 in increasing concentrations from 0.3 µg/ml to 3 µg/ml (Figure 3F). Increasing amounts of anti-TGF-β1 resulted in progressively decreasing quantities of active TGF-β1 produced. This effect was not due to the presence of anti-TGF-β1 in the luciferase assay because the blocking antibody was removed during the final washing, prior to incubation with fresh media for the luciferase assay. These results show that blockade of the stimulating dose of raTGF-β1 abrogated active TGF-β1 production.

To determine whether the TGF-β1 blocking antibody might block effects of the TGF-β1 produced by HCMV infected cells, EMT was first induced in uninfected HK-2 cells using raTGF-β1 for 48 hours, after which cells were washed to remove raTGF-β1, infected with HCMV at MOI of 1, and finally incubated with either media alone or media containing the TGF-β1 blocking antibody at 3 µg/ml. After 24 hours, cells were lysed, RNA extracted and RT-PCR performed for mRNAs induced in the PCR array. Results from cells incubated with the blocking antibody are shown as percent reduction compared to cells incubated with media alone (Figure S3). These results show that cells, after undergoing EMT and subsequently infected with HCMV, demonstrate 50–95% reduction of mRNA transcripts of molecules associated with EMT in the presence of the TGF-β1 blocking antibody, compared to similar cells in the absence of the TGF-β1 blocking antibody. This result suggests that the TGF-β1 produced by HCMV infected cells after EMT may have activity upon the producing cells (true autocrine activity), which is inhibited by the TGF-β1 blocking antibody.

### Effect of TGF-β1 and viral input upon TGF-β1 activation

**Figure 4:**
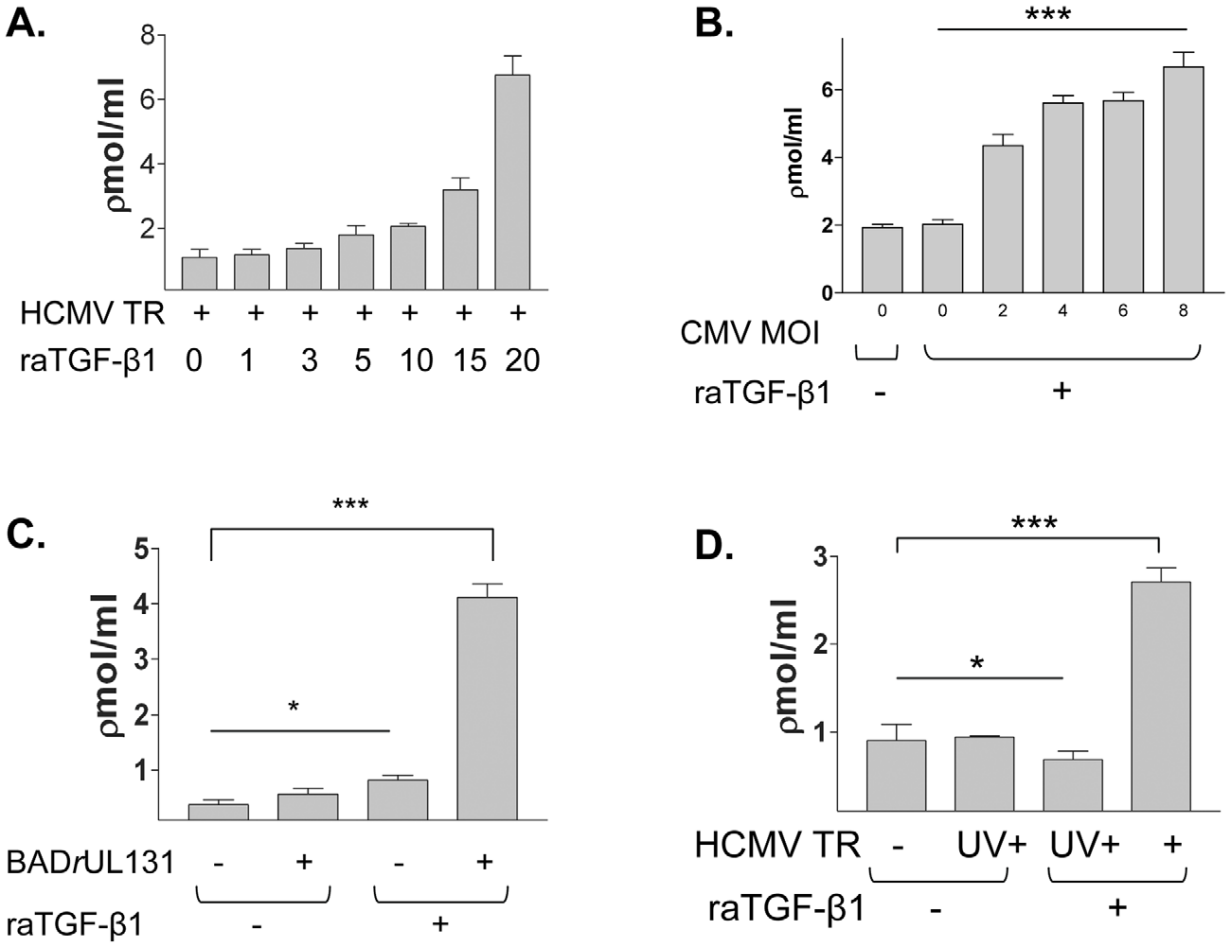
Effect of TGF-β1 and viral input upon TGF-β1 activation. (A) HK-2 cells were infected with HCMV strain TR at MOI of 1 and stimulated with increasing doses of raTGF-β1 from 1–20 ng/ml (0.04 nM–0.8 nM), washed, and assayed for *de novo* TGF-β1 production as described. HCMV infected HK-2 cells induced active TGF-β1 production in proportion to the amount of stimulating raTGF-β1. (B) HK-2 cells were infected with HCMV strain TR at increasing MOI (2-8) with raTGF-β1 stimulation at 15 ng/ml (0.6 nM), and luciferase bioassay performed to quantitate active TGF-β1 production. Active TGF-β1 production increased with increasing HCMV MOI. (C) HCMV AD169 strain BAD*r*UL131 at MOI of 1 was used to infect HK-2 cells prior to raTGF-β1 stimulation, and TGF-β1 luciferase bioassay performed. Similar to results for HCMV strain TR, HCMV AD169 strain BAD*r*UL131 induced active TGF-β1 production after EMT. (D) HCMV strain TR at MOI of 1 was inactivated by UV irradiation (HCMV TR UV+) and used to infect HK-2 cells prior to raTGF-β1 stimulation, and active TGF-β1 measured by luciferase bioassay. Irradiated virus failed to induce active TGF-β1 production after EMT. Legend: (*) p>0.05, ns; (***) p<0.01.

HCMV infected HK-2 cells were then stimulated with increasing amounts of raTGF-β1 at 0, 1, 3, 5, 10, 15, and 20 ng/ml, (0.04, 0.12, 0.2, 0.4, 0.6, 0.8 nM) and supernatants assayed for TGF-β1 activity using the luciferase bioassay. Active TGF-β1 increased with increasing stimulation dose (Figure 4A), whereas total TGF-β1 production under all conditions remained similar (dns). This result suggested that HCMV infected cells increased active TGF-β1 production in proportion to the quantity of TGF-β1 in the environment (autocrine production or auto-induction) [39]. Taken together, these results suggested that the quantity of TGF-β1 within an HCMV infected kidney might increase via autocrine production of additional TGF-β1 by HCMV infected epithelial cells undergoing EMT.

To determine whether the number of HCMV infected cells could influence the amount of active TGF-β1 produced, HK-2 cells were infected with HCMV strain TR at increasing multiplicity of infection (MOI) ranging from 2 to 10, stimulated with raTGF-β1 at 15 ng/ml (0.6 nM), and supernatants assayed for TGF-β1 production by luciferase bioassay. Increasing amounts of active TGF-β1 were produced by cells infected with HCMV at increasing MOI (Figure 4B). These results suggested that the level of active TGF-β1 increased with multiplicity of HCMV infection.

To determine whether TGF-β1 activation could be induced by HCMV strains other than TR, we utilized HCMV strain AD169 BAD*r*UL131 (kindly provided by T. Shenk, Princeton University, Princeton NJ), which contains a repaired mutation of the UL131 open reading frame (ORF), permitting infection of epithelial cells [40]. Wild-type AD169 and other HCMV strains commonly utilized *in vitro,* such as Towne, contain mutations in ORF UL128-131 which prevent infection of epithelial cells [40]. BAD*r*UL131 readily infected HK-2 cells as shown by immunofluorescent staining for IE1 antigen (dns), and was used to infect HK-2 cells with or without raTGF-β1 stimulation, followed by luciferase bioassay for TGF-β1 in the supernatants (Figure 4C). Similar to results for HCMV strain TR, BAD*r*UL131 alone did not induce TGF-β1 production (BAD*r*UL131+/raTGF-β1-), whereas infected cells stimulated by raTGF-β1 (BAD*r*UL131 +/raTGF-β1+) did induce active TGF-β1 production (p<0.01). Thus, induction of active TGF-β1 production by HCMV infected renal epithelium after EMT did not appear to be HCMV strain-specific.

To determine whether TGF-β1 production could be triggered by viral binding and entry into HK-2 cells without productive viral infection, HCMV strain TR was inactivated by ultraviolet irradiation (HCMV TR, UV+) and used to infect HK-2 cells. HCMV uninfected cells (HCMV TR -) and non-irradiated HCMV infected cells (HCMV TR +) served as negative and positive controls, respectively. Under these conditions, irradiated HCMV did not induce active TGF-β1 production after EMT (p>0.05), and did not affect total TGF-β1 levels either before or after EMT (Figure 4D). These results indicated that active TGF-β1 could not be induced solely by binding and entry of viral particles into cells undergoing EMT.

### A matrix metalloprotease complex is associated with TGF-β1 induction by HCMV infected cells after EMT

**Figure 5:**
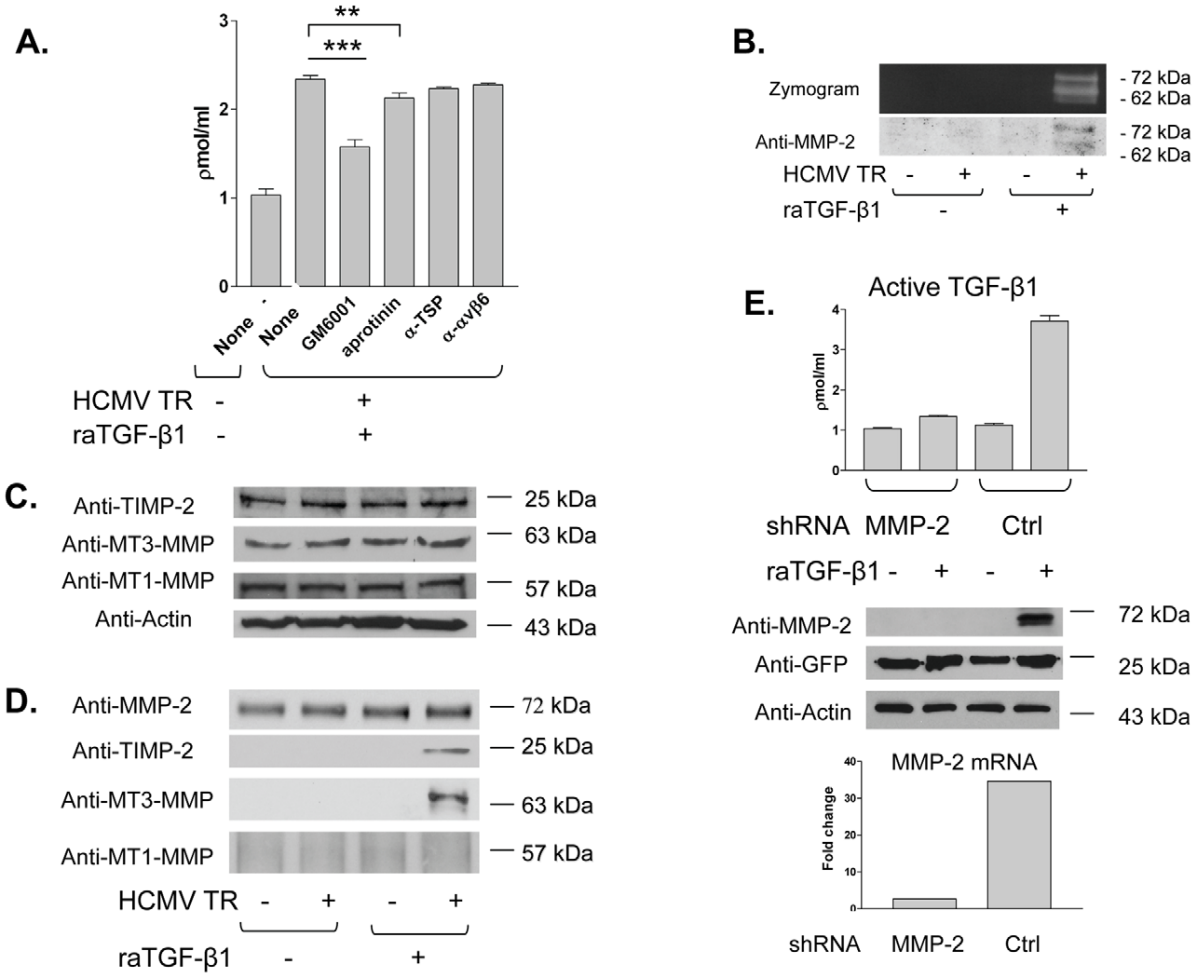
MMPs are expressed and a MMP complex forms in HCMV infected cells after EMT. (A) HK-2 cells were infected with HCMV strain TR at MOI of 1 and incubated with inhibitors, GM6001, aprotinin, anti-thrombospondin 1(α-TSP), or anti-α_v_β_6_ integrin (α- αvβ6), prior to stimulation with raTGF-β1 at 15 ng/ml (0.6 nM), washed, and TGF-β1 luciferase bioassay performed for active TGF-β1. Results were compared to those from uninfected, unstimulated HK-2 cells (HCMV TR-/raTGF-β1-) as well as HK-2 cells infected with HCMV and stimulated with raTGF-β1 (HCMV TR+/raTGF-β1+). Both GM6001 and aprotinin significantly inhibited active TGF-β1 production. Legend: (**) p<0.05; (***) p<0.01. (B) HK-2 cells were untreated, or were infected with HCMV at MOI of 1 and/or treated with raTGF-β1. Cell lysates were subjected to gelatin zymography (zymogram) and western blotting using anti-MMP-2 (anti-MMP-2). Pro- and active MMP-2 could be detected only in HCMV infected, raTGF-β1 stimulated cells. (C, D) HK-2 cells were treated as in (A), but lysates were either subjected directly to western blotting for TIMP-2, MT3-MMP, MT1-MMP, or actin (C) or incubated with mouse anti-MMP-2 followed by protein A-agarose, and immunoprecipitated proteins subjected to western blotting using rabbit anti-MMP-2, anti-TIMP-2, anti-MT3-MMP, and anti-MT1-MMP. TIMP-2 and MT3-MMP immunoprecipitated with MMP-2 only in HCMV infected, raTGF-β1 stimulated cells. (E) HK-2 cells were transfected with MMP-2 shRNA plasmid (MMP-2), or a control scrambled plasmid (Ctrl). Cells were infected with HCMV strain TR at MOI of 1 and/or stimulated with raTGF-β1 at 15 ng/ml. Supernatants were subjected to luciferase assay for active TGF-β1 (top panel). A portion of the cell pellets were subjected to western blotting for MMP-2, GFP, and actin (middle panel). RNA was extracted from the remainder of the cell pellets and RT-PCR performed for MMP-2 (bottom panel), with results depicted as fold change between raTGF-β1 exposed and non-exposed transfections. These assays showed that MMP-2 shRNA transfection reduced active TGF-β1, MMP-2 protein and mRNA; the control transfections stimulated with raTGF-β1 did induce active TGF-β1, MMP-2 protein and mRNA.

To characterize the mechanism by which HCMV infection might induce active TGF-β1 production, we added known inhibitors of TGF-β1 activation - aprotinin (serine protease inhibitor against plasmin), GM6001 (matrix metalloprotease inhibitor), anti-thrombospondin-1, anti-α_v_β_6_ integrin - to HCMV infected HK-2 cells prior to raTGF-β1 stimulation and again after washing, and the TGF-β1 luciferase bioassay was performed (Figure 5A). Active TGF-β1 production was reduced by approximately 60% (p<0.01) in the presence of GM6001. In the presence of aprotinin, active TGF-β1 production was decreased to a lesser but still statistically significant amount (19%, p<0.05). Active TGF-β1 was not significantly decreased in the presence of the other inhibitors, the blocking antibodies against thrombospondin 1 and the α_v_β_6_ integrin. This result suggested that MMPs and serine proteases might be involved in TGF-β1 activation by HCMV infected epithelial cells after EMT.

Because TGF-β1 is activated by MMP-2, we next analyzed MMP-2 production in our experimental conditions. Lysates from HK-2 cells with or without HCMV infection and/or raTGF-β1 stimulation were separated under non-reducing conditions by gelatin-containing SDS-PAGE and developed for presence of gelatinase activity. In Figure 5B, a representative zymogram showed bands at 72 and 62 kDa, consistent with pro- and active MMP-2, only in lysates from HCMV infected, raTGF-β1 stimulated cells (HCMV TR +/raTGF-β1 +). Identity of these bands as MMP-2 was confirmed by immunoblotting using anti-MMP-2 (Figure 5B). This result indicated that only HCMV infected, raTGF-β1 stimulated cells had cell-associated pro-MMP-2 and active MMP-2 as detected in this assay of functional MMP-2.

Since pro-MMP-2 is known to undergo activation in complex with other MMPs on the cell surface, we next performed immunoprecipitation experiments using lysates of HK-2 cells with or without HCMV infection and/or raTGF-β1 stimulation. Lysates were subjected to western blotting for TIMP-2, MT3-MMP, and MT1-MMP directly (Figure 5C), or were incubated with mouse anti-MMP-2, immune complexes collected with protein A-agarose, and Western blotting of immunoprecipitated proteins performed using a rabbit anti-MMP-2 antibody, or antibodies against TIMP-2, MT3-MMP, and MT1-MMP (Figure 5D). Immunoblotting for MMP-2 in the immunoprecipitates showed that MMP-2 was detectable in all samples after immunoprecipitation (Figure 5D). Immunoblotting of immunoprecipitated proteins demonstrated the presence of TIMP-2 and MT3-MMP only in the HCMV infected, raTGF-β1 treated cells (HCMV TR+/raTGF-β1+) (Figure 5D). MT1-MMP did not immunoprecipitate with anti-MMP-2 in any samples, and was not detectable even with membrane overexposure (Figure 5D). These results suggested that MMP-2 activation by HCMV infected, raTGF-β1 stimulated cells could occur via formation of a membrane-associated complex of MT3-MMP, TIMP-2, and MMP-2 [41].

To determine whether reduction of MMP-2 production could inhibit TGF-β1 activation, we next transfected a GFP-expressing shRNA construct against MMP-2 mRNA into HK-2 cells prior to HCMV infection and raTGF-β1 stimulation. A control plasmid, consisting of scrambled RNA serving to control for off-target effects, was transfected in parallel cultures. GFP expression was confirmed by fluorescence microscopy daily during the assay and confirmed similar transfection efficiencies. Active TGF-β1 in supernatants was evaluated by luciferase assay, and cell lysates were divided into equal portions and either assessed by western blotting for MMP-2, GFP, and actin, or RNA extracted for RT-PCR analysis of MMP-2. Active TGF-β1 production was inhibited by the MMP-2 shRNA construct but not the control irrelevant shRNA (Figure 5E, top panel). MMP-2 protein was not detectable in the samples transfected with the MMP-2 shRNA, but was detectable in the samples transfected with the control irrelevant shRNA treated with raTGF-β1 (Figure 5E, middle panel). GFP and actin expression were similar in all samples (Figure 5E, middle panel). RT-PCR analysis, depicted as a fold change between samples with and without raTGF-β1 exposure for either MMP-2 shRNA or the control shRNA, demonstrated detectable MMP-2 mRNA only in the control shRNA (Figure 5E, bottom panel). Taken together, these results indicate that reduction of MMP-2 production results in inhibition of TGF-β1 activation in this experimental system.

### HCMV IE1 and IE2 genes independently mediate TGF-β1 auto-induction

**Figure 6:**
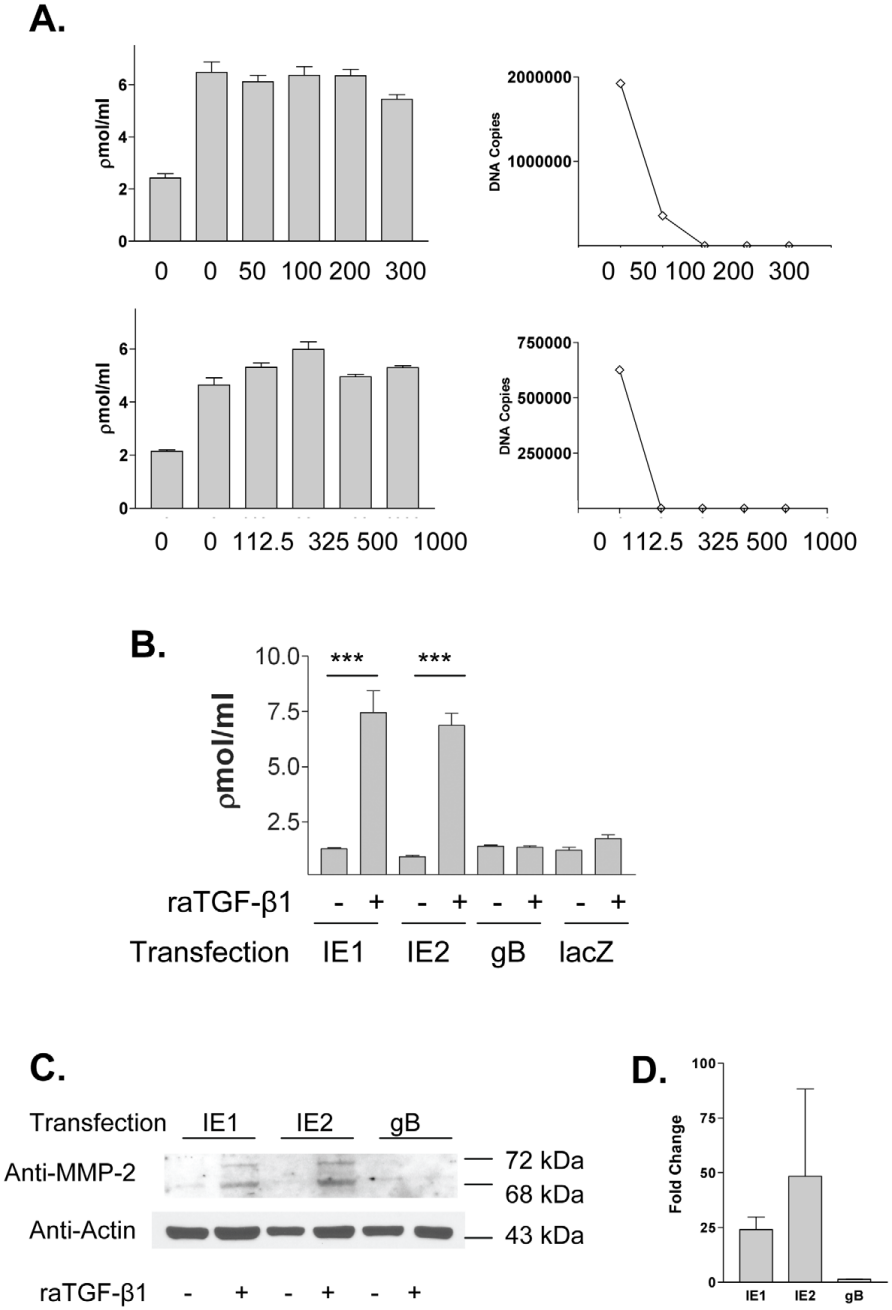
Effects of viral inhibitors and HCMV IE1 or IE2 ORFs upon TGF-β1 production. (A) HK-2 cells were infected with HCMV strain TR in the presence of increasing concentrations of ganciclovir or foscarnet prior to raTGF-β1 stimulation, and active TGF-β1 measured by luciferase bioassay (left panel). These inhibitors did not affect active TGF-β1 production by HCMV infected cells after EMT. DNA was extracted from cell pellets and quantitative DNA PCR for HCMV gB was performed (right panel), and confirmed efficacy of the viral polymerase inhibitors. (B) HK-2 cells were transiently transfected with expression plasmids containing lacZ either alone or co-transfected with plasmids containing either HCMV IE1, IE2, or UL55 (gB), followed by raTGF-β1 stimulation and TGF-β1 luciferase bioassay. Results from the TGF-β1 luciferase bioassay were normalized to transfection efficiency as measured by β-galactosidase activity in cell lysates (dns). Both the IE1 and IE2 constructs, but not the gB construct, induced active TGF-β1 production after EMT. Legend: (***) p<0.01. (C, D) Cells were transfected with plasmids containing either HCMV IE1, IE2, or UL55, and cell pellets were subjected to western blotting for MMP-2 and actin (C), or RT-PCR for MMP-2 mRNA (depicted as fold change between raTGF-β1 exposed and non-exposed transfections). Only the IE1 or IE2 transfected cells exposed to raTGF-β1 induced MMP-2 protein and mRNA.

To characterize the viral gene product requirements for TGF-β1 activation by HCMV infected epithelial cells after EMT, viral polymerase inhibitors ganciclovir (GCV) and foscarnet (PFA) were added at a range of concentrations to inhibit viral replication, one hour after HCMV TR infection but before raTGF-β1 stimulation, and again after washing, and the luciferase bioassay for TGF-β1 performed (Figure 6A, left panel). For each condition, DNA was extracted from cell lysates and quantitative DNA PCR for the HCMV UL55 ORF (gB) was performed (Figure 6A, right panel). Production of active and total TGF-β1 was not affected by either of the viral inhibitors, suggesting that the viral effect resulting in TGF-β1 production preceded viral replication as represented by viral polymerase inhibition. Together with results from experiments using irradiated viruses, this result suggested that TGF-β1 production after EMT and HCMV infection might involve the function of immediate early or early gene products.

To investigate the possible function of the major HCMV immediate early genes IE1 or IE2 in TGF-β1 activation, HK-2 cells were transiently transfected with expression plasmids containing the open reading frame for either IE1 or IE2, stimulated with raTGF-β1, and luciferase bioassay performed. A plasmid containing ORF UL55, encoding gB, was also transfected separately into HK-2 cells as a representative HCMV late gene product. Expression of transfected constructs was confirmed by immunofluorescent analysis for HCMV IE1, IE2, or gB antigens by cells grown on coverslips and by western blotting for IE1, IE2, or gB proteins in transfected cell lysates (dns). A lacZ-expressing plasmid was transfected separately or co-transfected with the other plasmids, and transfection efficiency was determined by quantitation of β-galactosidase activity in cell lysates. Results of the bioassay for active TGF-β1 were normalized to transfection efficiency. Plasmids containing either IE1 or IE2 did not induce TGF-β1 production before EMT, but did induce active TGF-β1 production after EMT, whereas control plasmids containing HCMV UL55 or lacZ did not induce TGF-β1 production either before or after EMT (Figure 6B). This result suggested that IE1 and IE2 might have a common transactivating function permitting TGF-β1 production by HCMV infected HK-2 cells after EMT.

Because MMP-2 was implicated in the TGF-β1 activating phenotype in HCMV infected cells (Figure 5), lysates from cells transfected with either IE1, IE2, or gB were divided equally and subjected to western blotting for MMP-2 or RT-PCR for MMP-2 mRNA. MMP-2 protein and mRNA were detectable in IE1 and IE2 transfections treated with raTGF-β1, but not in gB tranfections (Figure 6C, D). Immunoblotting for actin was performed as a loading control. These results are consistent with those from HCMV infected cells and suggest that the HCMV IE1/2 gene products may promote TGF-β1 activation via MMP-2 induction.

## Discussion

Epithelial-to-mesenchymal transition (EMT) is a well-characterized phenotypic change manifested by renal tubular epithelial cells after exposure to TGF-β1 and has been associated with various forms of renal fibrosis. TGF-β1 is present in renal allografts, including both human renal biopsies after transplantation, and in animal models of renal transplantation. TGF-β1 has been described to be more abundant in CMV infected renal allografts in both patient biopsies and animal transplantation models. However, the phenotype of CMV infected renal epithelial cells undergoing EMT has not previously been characterized.

In this study we have shown that, *in vitro*, HCMV infected renal tubular epithelial cells can undergo EMT after exposure to TGF-β1. After EMT, HCMV infected cells can induce extracellular activation of TGF-β1 via induction of MMP-2. This autocrine production of TGF-β1 could contribute to the observation in human and animal renal transplantation associating HCMV infection with increased expression of intra-renal TGF-β1. This autocrine process is thought to contribute to pathologic fibrosis *in vivo* [39]. Our *in vitro* findings are consistent with findings *in vivo* by Helantera et al., showing increased TGF-β1 in HCMV infected human renal biopsies as well as increased urinary TGF-β1 from patients with HCMV infected allografts [15], [42]. MMPs also participate in degradation of basement membrane, enhancement of cellular motility, activation of growth factors, and modulation of cell adhesion molecules, and have been implicated in various forms of renal fibrotic disease [43], [44]. Elevation of urinary MMPs has been described during renal allograft rejection but has not been explored in the context of HCMV infected renal allografts [45], [46]. Our finding that the complex of MMP-2, MT3-MMP, and TIMP-2 may contribute to TGF-β1 activation in HCMV infected HK-2 cells demonstrates that this ternary complex, which has been well described in vitro, may serve as a mechanism for TGF-β1 activation by HCMV infected renal tubular epithelial cells [41], [47], [48].

The concept that other factors (in this case, HCMV infection) might amplify the fibrogenic phenotype of HK-2 cells undergoing TGF-β1 induced EMT has been validated by others, who have shown that epidermal growth factor enhances the migratory phenotype of HK-2 cells and synergistically increases MMP-9 production after TGF-β1 induced EMT [49]. Interestingly, the fibrogenic phenotype enhanced by HCMV occurs only after EMT, as HCMV infected HK-2 cells in epithelial form did not manifest significant induction of TGF-β1. This suggests that HCMV infected renal epithelial cells, at baseline, do not induce fibrogenic renal changes, which would be consistent with the absence of primary renal pathology in asymptomatic humans infected with HCMV.

CMV effects upon extracellular matrix and fibrosis have been previously characterized in the context of rat CMV infection of renal and cardiac allografts. In those models, rat CMV intensified production of fibrogenic molecules such as TGF-β1, PDGF, and collagens in renal allografts, and was associated with transcriptional upregulation of numerous fibrogenic and angiogenic molecules in the cardiac allograft [7], [17], [50]. HCMV infection has also been shown to induce TGF-β1 activation by endothelial cells via an integrin-mediated mechanism, suggesting that placental infection by HCMV may alter extracellular matrix and permit HCMV translocation across the placenta, contributing to congenital infection of the fetus [31]. Our work, the first to investigate HCMV infection of renal tubular cells *in vitro*, supports the findings by others that HCMV may modify the extracellular matrix during inflammatory conditions such as solid organ transplantation or chorioamnionitis.

We have also shown that, similar to fibroblasts and astrocytes, transient transfection of plasmids encoding the HCMV IE1 or IE2 gene products can induce TGF-β1 activation via MMP-2 by renal epithelial cells after EMT. The inability of viral DNA polymerase inhibitors, ganciclovir and foscarnet, to affect TGF-β1 activation after EMT in HCMV infected epithelial cells supports the role of gene products from IE1 and/or IE2 in this phenotype, and suggests potential limited utility of these antivirals in preventing HCMV associated fibrosis in the clinical setting. Interestingly, the promoters for IE1, IE2, and numerous MMPs all contain AP-1 binding sites, and in the case of MMPs, this transcription factor is thought to contribute to control of transcriptional upregulation [51].

Our finding that either the IE1 or IE2 gene products may be sufficient to induce TGF-β1 production suggests that HCMV reactivation within the transplanted kidney may be associated with the pathogenesis of HCMV associated renal allograft damage. These conditions would be present uniquely in renal allografts, with host T cell immunosuppression and local allograft inflammation permitting HCMV reactivation within the allograft, and presence of local TGF-β1 in the allograft inducing EMT [34], [52], [53]. These conditions would all exist simultaneously after renal transplantation, but not necessarily in other forms of fibrotic renal disease in which TGF-β1 may be present.

In summary, this *in vitro* model shows that HCMV infected renal tubular epithelial cells undergo EMT after exposure to TGF-β1. HCMV infection may amplify autocrine TGF-β1 production via an MMP cascade. The HCMV IE1/IE2 transcription factors are each capable of inducing active TGF-β1 production, suggesting that clinically utilized antivirals might not prevent this HCMV effect within the kidney. These *in vitro* studies provide a potential pathogenic mechanism for the observed association between HCMV infection, TGF-β1 production, and poor clinical allograft outcome in human renal transplant recipients.

## Materials and Methods

### Cells and viruses

The immortalized human renal proximal tubular epithelial cell line, HK-2, was purchased from American Type Culture Collection (Manassas, VA) and maintained in keratinocyte serum-free media (Invitrogen, Carlsbad CA). Primary human renal proximal tubular epithelial cells (Lonza, Walkersville MD) were maintained in renal epithelial growth media (Lonza). Mink lung epithelial cells stably expressing the TGF-β response element of the plasminogen activator inhibitor-1 promoter fused to the firefly luciferase reporter gene (gift of D. Rifkin, New York University, New York NY) were maintained as described [54]. Primary human foreskin fibroblasts (HFFs) were maintained as described [55].

HCMV strain TR (gift of J. Nelson, Oregon Health and Sciences University, Portland OR) and BAD*r*UL131 (gift of T. Shenk, Princeton University, Princeton NJ) were propagated in HFFs. Viruses were concentrated by centrifugation at 16,000×g for 2 hours at 4°C, resuspended in keratinocyte serum-free media, and frozen at −80°C until use. For ultraviolet inactivation, HCMV was exposed to ultraviolet radiation at 150 mJ in a cross-linking chamber (Bio-Rad, Hercules CA) [56]. Virus titers were determined using a standard assay for detection of early antigen fluorescent foci (DEAFF) in fibroblasts [57], [58].

### Chemicals, antibodies, and reagents

The following reagents were purchased from commercial vendors: recombinant human active TGF-β1 (raTGF-β1), Quantikine human TGF-β1 ELISA, TGF-β1 blocking antibody (clone 9016) (R&D Systems, Minneapolis MN); luciferase assay reagent, β-galactosidase assay kit (Promega Corp., Madison WI); RNeasy kit (Qiagen, Valencia CA); RT^2^ First Strand Kit, SuperArray Human Extracellular Matrix PCR Array, human MMP-2 shRNA kit (SABiosciences, Frederick MD), Cells-to-CT kit (Applied Biosystems, Foster City CA); GM6001, rabbit anti-MMP-2 antibody (AB19167); rabbit anti- MT3-MMP antibody (AB853), mouse anti-α_v_β_6_ blocking antibody (MAB2077Z) (Millipore, Billerica MA); pEF1/myc-his/lacZ plasmid, anti-GFP antibody, AlexaFluor conjugated phalloidin and secondary antibodies, *and* SuperScript III kit (Invitrogen); Cytogam (CSL Behring, King of Prussia PA); Nucleofector device and transfection kit V (Amaxa, Gaithersburg MD). The following human primer/probe sets were purchased from ABI: fibronectin (Hs.01549976_m1); MMP-9 (Hs.00957562_m1); ADAMTS1 (Hs.00199608_m1); TGF-β1 (Hs.00932734_m1); collagen 5A1 (Hs.00609088_m1); MMP-2 (Hs.01548733_m1); thrombospondin-1 (Hs.00170236_m1); 18S RNA (part #4333760-0904029). Mouse anti-MMP-2 (mab CA801), mouse anti-TIMP-2 (mab101), and rabbit anti-MT1-MMP polyclonal antisera (pab 198) were a kind gift from R. Fridman (Wayne State University, Detroit MI). Ganciclovir and foscarnet were kindly provided by M. Prichard (University of Alabama-Birmingham, Birmingham AL). Anti-thrombospondin 1 antibody (mab133), mouse monoclonal antibodies against HCMV IE1, IE2, and gB (mab 63-27, mab 2-9-5, and mab 7-17), and expression plasmids containing HCMV IE1, IE2, or UL55 open reading frames were provided by the authors (J. M.-U. and W. B.).

### Viral titration

Primary HFFs, HK-2 cells, or primary renal tubular epithelial cells were grown in 6-well plates, infected with HCMV at an MOI of 1 for one hour, washed and incubated with fresh media, and cells and media harvested daily for 6 days and stored at −80°C. Cells were resuspended in 500 µl DPBS, sonicated briefly, serial dilutions made, and DEAFF assay performed. Serial dilutions of media were also analyzed by DEAFF assay.

### Immunofluorescent staining of HCMV infected cells

Cells were grown on coverslips, infected with HCMV TR and/or incubated with recombinant active TGF-β1 (raTGF-β1) for 48 hours, fixed in 4% paraformaldehyde, and permeabilized using 0.1% Triton X-100. Cells were incubated with primary antibodies overnight at 4°C, washed, incubated for 1 hour with isotype and species specific secondary antibodies labeled with either AlexaFluor 488 or AlexaFluor 594 followed by Topro3 nuclear stain and mounted using ProLong anti-fade reagent. Images were collected under similar exposure times and identical gain using confocal fluorescence microscopy (Olympus Fluoview BX51, Center Valley PA).

### Immunoblotting assays

HK-2 cells were untreated, or infected with HCMV TR and/or treated with raTGF-β1 at 15 ng/ml (0.6 nM). Cells were washed, pelleted by centrifugation, lysed, and equivalent protein lysates were boiled and subjected to 10% SDS-PAGE and immunoblotting with enhanced chemiluminescence as described [59]. Immunoblots were analyzed using Adobe Photoshop densitometry software (Adobe Systems Inc, San Jose CA). For phospho-SMAD2 immunoblotting, cells were harvested at 1 hour after raTGF-β1 exposure, lysed and nuclear extracts prepared as described [60]. Nuclear proteins were separated by SDS-PAGE, transferred to nitrocellulose, and immunoblotting performed as above.

### Reverse-transcriptase PCR of HCMV infected cells

HK-2 cells were grown in T75 flasks, either untreated or infected with HCMV TR and/or incubated with raTGF-β1 for 24 hours, lysed using TriPure reagent, and RNA extracted using the RNeasy kit. RNA was reverse transcribed to cDNA using the RT^2^ First Strand Kit, and cDNAs used according to the manufacturer's instructions for the SuperArray Human Extracellular Matrix PCR Array. Transcripts were normalized by comparison to GAPDH cDNA, and samples from experimental conditions were quantitated as fold-change compared to baseline production in uninfected, unstimulated HK-2 cells. For RT-PCR using commercial primer/probe sets (ABI), HK-2 cells or primary renal tubular epithelial cells were prepared as described and RNA extracted using the RNeasy kit. RNA was reverse transcribed to cDNA using the SuperScript III Kit. Reactions were performed in triplicate in 20 µl volumes consisting of ABI 2x MasterMix (10 µl), ABI primer/probe mix (1 µl), template (1 µl), and water (8 µl) and real-time PCR performed according to the manufacturer's instructions. Results were normalized to 18S RNA and depicted as fold change from baseline (uninfected, unstimulated cells). Experiments were performed three separate times.

### Assays for TGF-β1 production

HK-2 cells or primary renal tubular epithelial cells were seeded onto 24 well plates (2×10^4^ cells/well) or 6 well plates (1×10^5^ cells/well), either untreated or infected with HCMV at an MOI of 1 (strain TR, BAD*r*UL131, or UV inactivated TR) and/or treated with raTGF-β1 for 48 hours to induce EMT. For the CMV MOI assay, HCMV strain TR was added at an MOI of 2, 4, 6, 8, or 10 to each well. Wells were then washed three times with media to remove all raTGF-β1 and re-incubated with fresh media not containing raTGF-β1. Supernatants were collected at 24 hours post wash and stored at −80°C until assayed for TGF-β1 production. For the luciferase bioassay, supernatants were assayed for active and total TGF-β production using the mink lung epithelial cell reporter bioassay as described [54]. TGF-β concentrations (ρmol/ml) were calculated by comparison to a standard curve derived from known quantities of raTGF-β1. The Quantikine human TGF-β1 ELISA and the Ebioscience ELISA were used to quantitate active and total TGF-β1 according to the manufacturer's instructions. Some studies were performed in parallel using samples from the same experiments for both luciferase assays and ELISA for direct comparison of both assays.

In some experiments, HK-2 cells were untreated or infected with HCMV TR at MOI of 1 and/or stimulated with raTGF-β1 at 15 ng/ml (0.6 nM). The TGF-β1 blocking antibody was added at 0.3, 1, 2, and 3 µg/ml prior to addition of raTGF-β1 but was not re-added after washing and addition of fresh media for luciferase assay.

Next, HK-2 cells were stimulated to undergo EMT by exposure to raTGF-β1 for 48 hours. Cells were washed three times with media to remove exogenous raTGF-β1, then were infected with HCMV at MOI of 1. Cells were either incubated with media alone, or with media containing the TGF-β1 blocking antibody at 3 µg/ml for 24 hours. Cells were washed, lysed and total RNA extracted using the RNeasy kit. Reverse transcription assays were performed as described, and cDNA used as template for real-time PCR assays as described. Results from samples incubated with the TGF-β1 blocking antibody were compared to those from samples without the blocking antibody (baseline), and differences in mRNA expression depicted as percent reduction from baseline.

### Assays for inhibition of TGF-β1 activation

HK-2 cells were untreated or infected with HCMV TR at MOI of 1 and/or stimulated with raTGF-β1 at 15 ng/ml (0.6 nM). TGF-β1 inhibitors were added to wells one hour prior to raTGF-β1 stimulation at the following concentrations: GM6001 at 0.5 nM; aprotinin at 200 µg/ml; anti-thrombospondin-1 at 25 ng/ml; anti-α_v_β_6_ at 10 µg/ml [61], [62], [63], [64]. After 48 hours, cells were washed, inhibitors re-added, supernatants harvested at 24 hours post-wash, and luciferase bioassay performed.

### Zymography

HK-2 cells were untreated or infected with HCMV TR at MOI of 1 and/or treated with raTGF-β1 at 15 ng/ml (0.6 nM). Cells were washed, pelleted by centrifugation, lysed, and equivalent protein lysates were subjected to gelatin zymography as described [65]. Gels were stained with Coomassie Blue and photographed using the Quantity One gel imaging system (Bio-Rad).

### Co-Immunoprecipitation with anti-MMP-2

HK-2 cells were untreated, or infected with HCMV TR at MOI of 1 and/or stimulated with raTGF-β1 as described. Cells were washed, pelleted by centrifugation, lysed in cold RIPA buffer with protease inhibitors, incubated at 4°C overnight with anti-MMP-2, followed by incubation with protein A-agarose, washed and resuspended in RIPA buffer, boiled, and separated by 8% SDS-PAGE under reducing conditions. An aliquot of the original cellular lysate was saved prior to immunoprecipitation. Western blotting was performed for the original lysate and the immunoprecipitated material as described using antibodies against TIMP-2, MT1-MMP, and MT3-MMP.

### MMP-2 shRNA transfection

HK-2 cells were transfected with a commercial MMP-2 shRNA construct or a control scrambled construct provided by the manufacturer (SABiosciences), using the Amaxa Nucleofector kit V and Nucleofector program T-020 according to the manufacturer's instructions. Tranfected cells were each divided into two separate wells and observed daily by fluorescence microscopy for GFP expression. Wells were infected with HCMV strain TR at MOI of 1, and one of each pair were stimulated with raTGF-β1, washed, and supernatants and cell pellets harvested. Supernatants were assayed for TGF-β1 production by luciferase assay. Cell pellets were divided into equal portions, half of which was subjected to western blotting for MMP-2, GFP, and actin. The other half underwent total RNA extraction using the Cells to CT kit, and RT-PCR was performed using primer/probe sets for MMP-2 and 18S RNA according to the manufacturer's instructions (ABI). Results were normalized to 18S mRNA expression and depicted as fold change between samples treated with and without raTGF-β1 for each shRNA transfection condition.

### Assays for antiviral inhibition of TGF-β1 activation

HCMV TR infected HK-2 cells on coverslips were incubated with ganciclovir at 50, 100, 200, and 300 µM, foscarnet at 112.5, 325, 500, and 1000 µM, stimulated with raTGF-β1 at 15 ng/ml (0.6 nM), washed and re-incubated with inhibitors, supernatants harvested 24 hours after washing and luciferase bioassay performed [66], [67], [68]. Cells were harvested separately, DNA extraction performed using the Qiagen DNA Blood kit, and quantitative DNA PCR performed using primers and probe for a highly conserved sequence within HCMV gB and compared to a standard curve generated by real-time PCR amplification of known copy numbers (10^1^–10^8^ copies) of a plasmid containing the gB DNA sequence recognized by the gB primer/probe pair [69]. Results were depicted as DNA copies based upon comparison with the plasmid standard curve.

### Transient transfection assays

In 6-well plates containing coverslips, 1×10^6^ HK-2 cells were transfected with the expression plasmid containing lacZ either alone or with expression plasmids containing either IE1, IE2, or UL55, using the Nucleofector kit V and Nucleofector program T-020. At 24 hours post transfection, some wells were stimulated with raTGF-β1 at 15 ng/ml (0.6 nM) for 48 hours, washed, and all supernatants assayed for TGF-β1 by luciferase bioassay. Coverslips were fixed and stained as described for IE1, IE2, or gB protein expression. Cells were lysed and assayed for β-galactosidase activity according to the manufacturer's instructions using an ELISA microplate reader (ELx808, Bio-Tek Instruments, Inc., Winooski, VT). Results from the luciferase bioassay were normalized to relative transfection efficiency as determined by β-galactosidase expression.

In a separate experiment, transfections and raTGF-β1 stimulation were performed as described, and cell lysates were separated into two aliquots and subjected either to western blotting for MMP-2 protein or RT-PCR for MMP-2 mRNA expression as described. Results were depicted as a fold change for each transfection condition between unstimulated and raTGF-β1 stimulated cells.

### Statistical analysis

All assays were performed with triplicate samples and results expressed as mean ± SEM. The Student T test and one-way analysis of variance (ANOVA) were used to compare groups using Prism 3.0 software, accepting statistically significant differences at a p value of<0.05 (GraphPad, San Diego CA). All experiments, except the PCR array, were performed at least three times independently to confirm the reproducibility of each result.

## Supporting Information

Figure S1 HCMV replication in primary renal tubular epithelial cells. Primary renal tubular epithelial cells were infected with HCMV strain TR at MOI of 1, cells and media harvested daily, and viral titers determined by DEAFF assay. These primary renal epithelial cells supported linear productive infection in cell pellets, similar to HK-2 cells. Although a few virions could be detected in the media over time, the quantity did not consistently increase over time. Upright triangles, cells; inverted triangles, media.
(0.06 MB TIF)

Figure S2 HCMV infected HK-2 cells and renal tubular epithelial cells express mRNA transcripts suggestive of epithelial-to-mesenchymal transition (EMT) after TGF-β1 exposure. HK-2 cells (A) or primary renal tubular epithelial cells (B) were untreated, or were infected with HCMV strain TR at MOI of 1 and/or stimulated with raTGF-β1 to induce EMT, lysed, total RNA reverse transcribed to cDNA, and cDNA analyzed for presence of extracellular matrix associated mRNAs using commercial primer/probe pairs designed to detect cDNA but not genomic DNA for the target of interest. Results were normalized to 18S mRNA expression and quantitated as fold-change compared to baseline mRNA levels in uninfected, unstimulated cells. Results from HCMV infected cells (grey bars), uninfected cells stimulated with raTGF-β1 (hatched bars), and HCMV infected cells stimulated with raTGF-β1 (black bars) confirmed similar induction of the fibrogenic molecules shown to be upregulated in the PCR array after exposure to raTGF-β1 (Figure 2C). Although the degree of induction was lower for some transcripts (MMP-9, ADAMTS1) in the primer/probe assay compared to the results from the PCR array, overall these results suggest that HCMV infected HK-2 cells and primary renal tubular epithelial cells after raTGF-β1 stimulation do express transcripts consistent with induction of EMT.
(0.19 MB TIF)

Figure S3 A TGF-β1 blocking antibody reduces EMT-associated mRNA transcripts in HCMV infected HK-2 cells. HK-2 cells were stimulated to undergo EMT by exposure to raTGF-β1 for 48 hours. Cells were washed three times with media to remove exogenous raTGF-β1, then were infected with HCMV at MOI of 1. Cells were then either incubated with media alone, or with media containing a TGF-β1 function blocking antibody at 3 µg/ml for 24 hours. Cells were washed, lysed, total RNA extracted and reverse transcribed to cDNA, and real-time PCR assays performed using commercial primer/probe sets. Results from samples incubated with the TGF-β1 blocking antibody were compared to those from samples without the blocking antibody (baseline), and differences in mRNA expression depicted as percent reduction from baseline. These results show a reduction in mRNA transcripts for these molecules in the presence of the TGF-β1 blocking antibody, suggesting that blockade of the activity of TGF-β1 produced by the HCMV infected cells may reduce transcription of these mRNAs. Legend: TSP-1, thrombospondin-1.
(0.07 MB TIF)
