# Supplementary figures and images for: Human Cytomegalovirus Induces TGF-β1 Activation in Renal Tubular Epithelial Cells after Epithelial-to-Mesenchymal Transition
Source: PLoS Pathog. 2010 Nov 4;6(11):e1001170. doi: 10.1371/journal.ppat.1001170 (PMC2973835; doi:10.1371/journal.ppat.1001170)

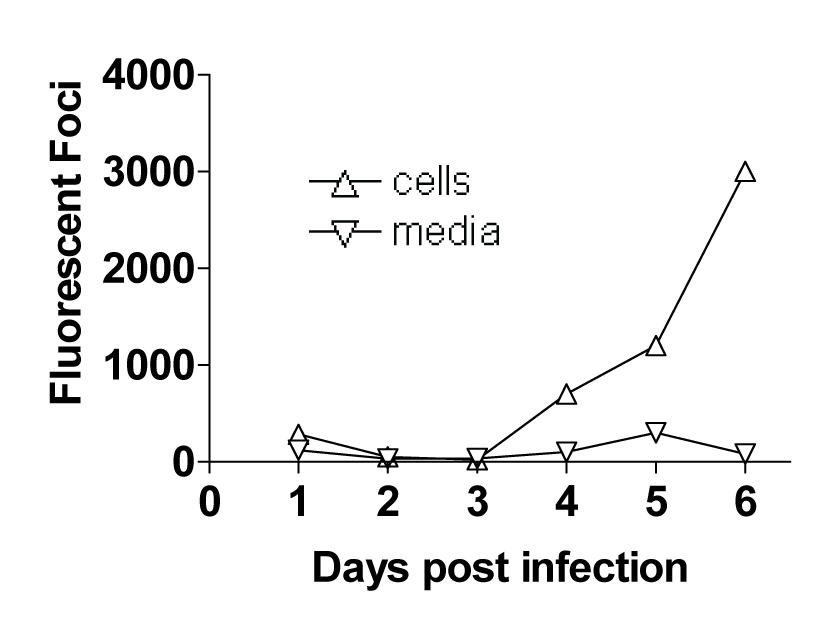

Supplement: Figure S1 — HCMV replication in primary renal tubular epithelial cells. Primary renal tubular epithelial cells were infected with HCMV strain TR at MOI of 1, cells and media harvested daily, and viral titers determined by DEAFF assay. These primary renal epithelial cells supported linear productive infection in cell pellets, similar to HK-2 cells. Although a few virions could be detected in the media over time, the quantity did not consistently increase over time. Upright triangles, cells; inverted triangles, media. (0.06 MB TIF) [file ppat.1001170.s001.tif]

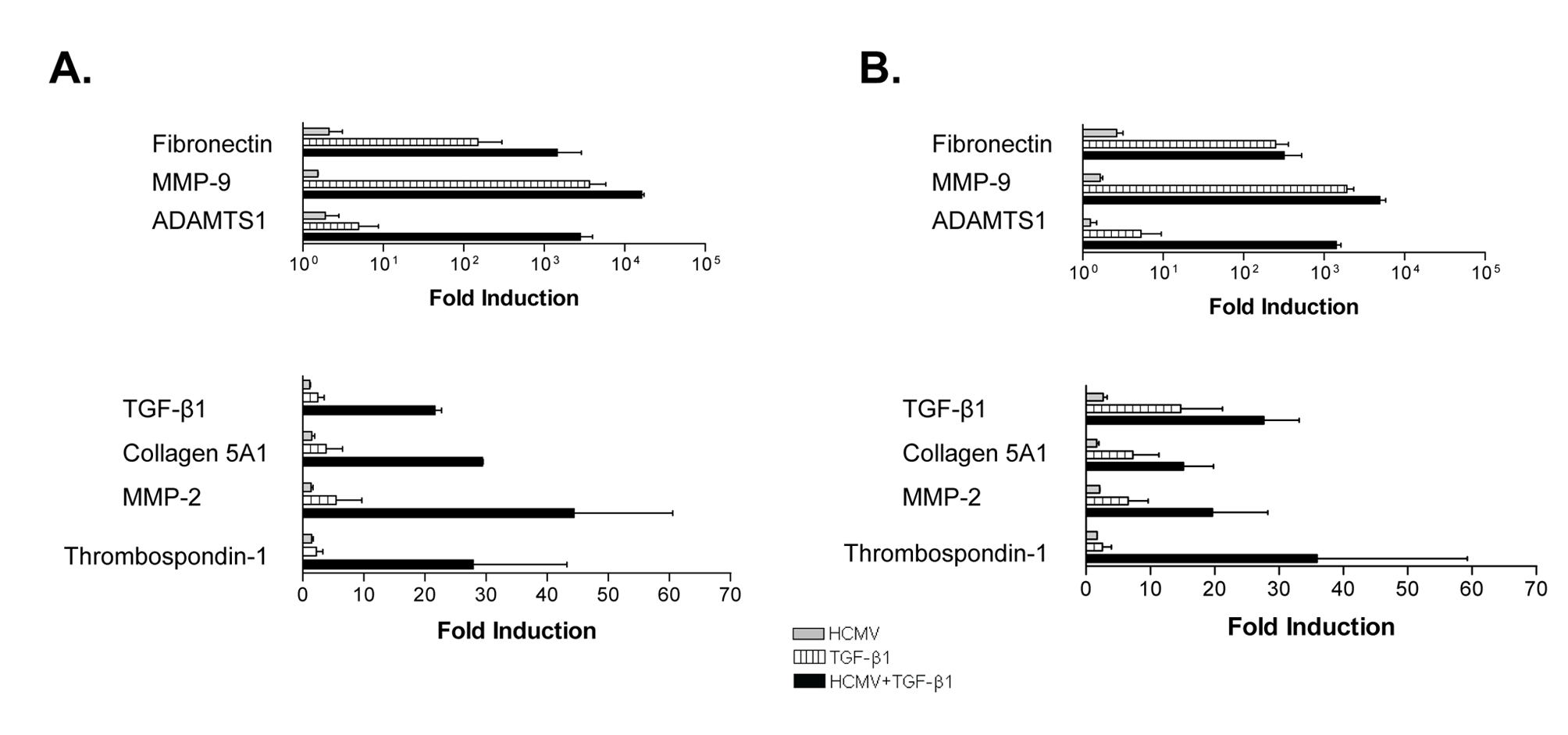

Supplement: Figure S2 — HCMV infected HK-2 cells and renal tubular epithelial cells express mRNA transcripts suggestive of epithelial-to-mesenchymal transition (EMT) after TGF-β1 exposure. HK-2 cells (A) or primary renal tubular epithelial cells (B) were untreated, or were infected with HCMV strain TR at MOI of 1 and/or stimulated with raTGF-β1 to induce EMT, lysed, total RNA reverse transcribed to cDNA, and cDNA analyzed for presence of extracellular matrix associated mRNAs using commercial primer/probe pairs designed to detect cDNA but not genomic DNA for the target of interest. Results were normalized to 18S mRNA expression and quantitated as fold-change compared to baseline mRNA levels in uninfected, unstimulated cells. Results from HCMV infected cells (grey bars), uninfected cells stimulated with raTGF-β1 (hatched bars), and HCMV infected cells stimulated with raTGF-β1 (black bars) confirmed similar induction of the fibrogenic molecules shown to be upregulated in the PCR array after exposure to raTGF-β1 (Figure 2C). Although the degree of induction was lower for some transcripts (MMP-9, ADAMTS1) in the primer/probe assay compared to the results from the PCR array, overall these results suggest that HCMV infected HK-2 cells and primary renal tubular epithelial cells after raTGF-β1 stimulation do express transcripts consistent with induction of EMT. (0.19 MB TIF) [file ppat.1001170.s002.tif]

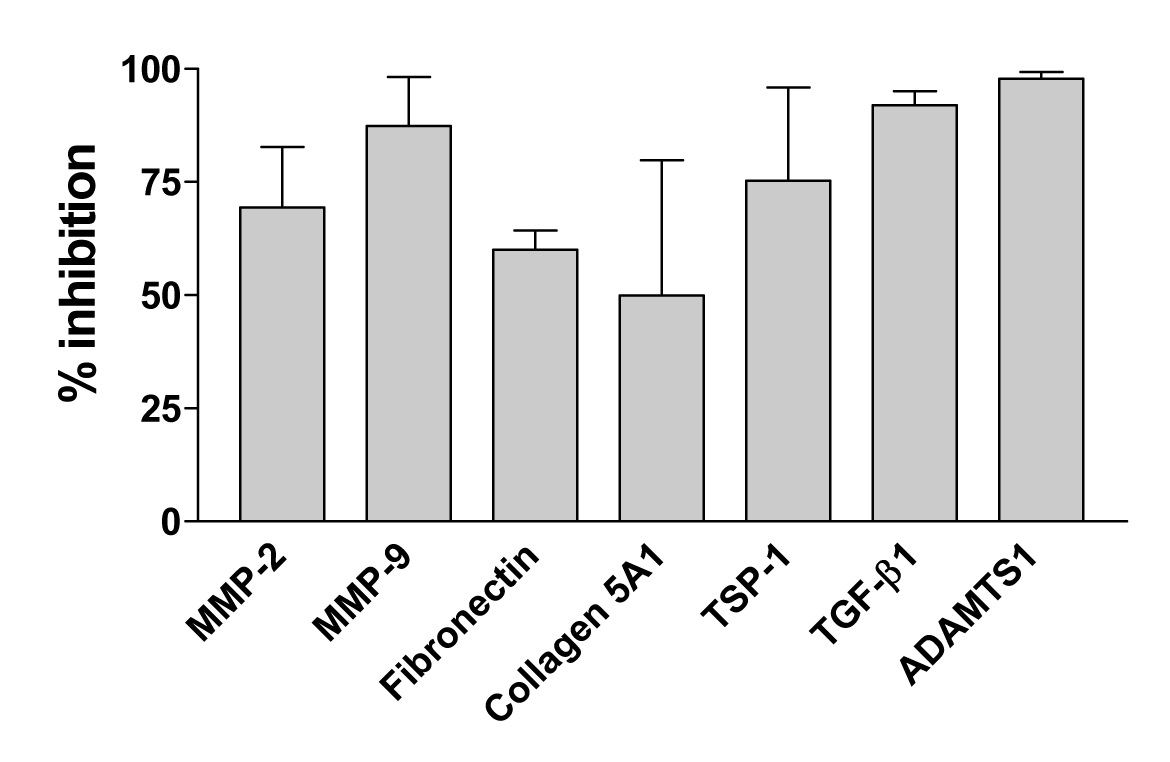

Supplement: Figure S3 — A TGF-β1 blocking antibody reduces EMT-associated mRNA transcripts in HCMV infected HK-2 cells. HK-2 cells were stimulated to undergo EMT by exposure to raTGF-β1 for 48 hours. Cells were washed three times with media to remove exogenous raTGF-β1, then were infected with HCMV at MOI of 1. Cells were then either incubated with media alone, or with media containing a TGF-β1 function blocking antibody at 3 µg/ml for 24 hours. Cells were washed, lysed, total RNA extracted and reverse transcribed to cDNA, and real-time PCR assays performed using commercial primer/probe sets. Results from samples incubated with the TGF-β1 blocking antibody were compared to those from samples without the blocking antibody (baseline), and differences in mRNA expression depicted as percent reduction from baseline. These results show a reduction in mRNA transcripts for these molecules in the presence of the TGF-β1 blocking antibody, suggesting that blockade of the activity of TGF-β1 produced by the HCMV infected cells may reduce transcription of these mRNAs. Legend: TSP-1, thrombospondin-1. (0.07 MB TIF) [file ppat.1001170.s003.tif]
